# Embodied Object Representation Learning and Recognition

**DOI:** 10.3389/fnbot.2022.840658

**Published:** 2022-04-14

**Authors:** Toon Van de Maele, Tim Verbelen, Ozan Çatal, Bart Dhoedt

**Affiliations:** IDLab, Department of Information Technology, Ghent University - imec, Ghent, Belgium

**Keywords:** generative modeling, robotic perception, deep learning, active inference, representation learning

## Abstract

Scene understanding and decomposition is a crucial challenge for intelligent systems, whether it is for object manipulation, navigation, or any other task. Although current machine and deep learning approaches for object detection and classification obtain high accuracy, they typically do not leverage interaction with the world and are limited to a set of objects seen during training. Humans on the other hand learn to recognize and classify different objects by actively engaging with them on first encounter. Moreover, recent theories in neuroscience suggest that cortical columns in the neocortex play an important role in this process, by building predictive models about objects in their reference frame. In this article, we present an enactive embodied agent that implements such a generative model for object interaction. For each object category, our system instantiates a deep neural network, called Cortical Column Network (CCN), that represents the object in its own reference frame by learning a generative model that predicts the expected transform in pixel space, given an action. The model parameters are optimized through the active inference paradigm, i.e., the minimization of variational free energy. When provided with a visual observation, an ensemble of CCNs each vote on their belief of observing that specific object category, yielding a potential object classification. In case the likelihood on the selected category is too low, the object is detected as an unknown category, and the agent has the ability to instantiate a novel CCN for this category. We validate our system in an simulated environment, where it needs to learn to discern multiple objects from the YCB dataset. We show that classification accuracy improves as an embodied agent can gather more evidence, and that it is able to learn about novel, previously unseen objects. Finally, we show that an agent driven through active inference can choose their actions to reach a preferred observation.

## 1. Introduction

Having a machine understand the world from pixels has been a long standing challenge defining the field of computer vision (Hanson, 1978). In the last decade, we have witnessed a proliferation of deep learning techniques in this domain, which started with the leap in performance obtained by a convolutional neural network (CNN) on object classification (Krizhevsky et al., 2012). Besides the exponential scaling of available compute resources, this progress is mainly fueled by the collection of massive datasets like ImageNet (Deng et al., 2009). The main strength of these techniques is that their classification accuracy typically improves as they are trained on more data, scaling to datasets containing billions of images (Mahajan et al., 2018). However, this strength is also becoming a main point of critique, as an exponential increase in compute (and energy) resources is required for marginal gains (Thompson et al., 2021). Moreover, these classifiers are known to be vulnerable to ambiguous and adversarial samples (Gilmer et al., 2018), and are restricted to object categories known and seen during training.

Humans on the other hand are embodied agents (Safron, 2021), allowing them to resolve ambiguity by actively sampling the world (Mirza et al., 2018). They are also much better learners: by the age of two, toddlers can recognize around 300 object categories (Frank et al., 2016), and can generalize a newly learned label to instances they have never seen before (Landau et al., 1988). Moreover, toddlers actively engage with their environment, visually exploring objects from various viewpoints by looking at and playing with them (James et al., 2014). In contrast to datasets collected for machine learning, which aim to collect a large and diverse set of exemplars of each object category, toddlers rather learn from a severely skewed data distribution, where only a small set of object instances are pervasively present, yet still we are able to generalize (Clerkin et al., 2017). Therefore, we propose a more enactive method for object category learning, in which an artificial agent can actively sample viewpoints.

Predictive coding is a paradigm based on the hypothesis of the Bayesian brain (Rao and Ballard, 1999), which makes the assumption that cortical circuits perform Bayesian inference to find the hidden causes of the observed signals. According to this paradigm, the brain entails a generative model and uses this to encode the error on the predicted observation.

Active inference is a process theory of sentience, which states that intelligent systems build a generative model of their world and act by minimizing a bound on surprise, i.e., the variational free energy (Friston et al., 2016). As such, active inference can not only be used to build artificial agents (Çatal et al., 2020a), but also to develop theories about functioning of the brain (Parr and Friston, 2018). For instance, Parr et al. (2021) propose an active inference account for human vision, which considers perception as inferring a scene as a factorization of separate (parts of) objects, their identity, scale and pose. Factorizing object identify from their scale and pose is consistent with the so called two stream hypothesis, which states that visual information is processed by a dorsal (“where”) stream on the one hand, representing where an object is in the space, and a ventral (“what”) stream on the other hand, representing object identity (Mishkin et al., 1983).

Similarly, Hawkins et al. (2017) hypothesize that cortical columns in the neocortex build object-centric models, capturing their pose in a local reference frame, encoded by cortical grid cells. Also empirical evidence from cognitive psychology showed that humans, given a single view of an object never seen before, have strong expectations about rotated views of that object, implying internal representations of three dimensional objects rather than two dimensional views (Tse, 1999). Recent findings in recordings of rhesus monkey brains provide evidence that indeed 3D shape is encoded in the inferior temporal cortex (Janssen et al., 2000).

Drawing inspiration from all these findings, we present a system for learning object-centric representations from pixel data. Akin to how a toddler interacts with a toy, we devise an artificial agent that can look at a 3D object from different viewpoints in a simulated environment. Parallel to cortical columns, our system learns separate models, which we call Cortical Column Networks (CCN) for separate object categories, which encode object pose and identity in two separate factors. An ensemble of CCNs then forms the agent's generative model, which is optimized by minimizing free energy. By engaging in active inference, our agent can realize preferred viewpoints for certain objects, while also resolving ambiguity on object identity.

Building on previous work (Van de Maele et al., 2021a), we now evaluate our agent on pixel data rendered from 33 objects from the YCB benchmarking dataset (Calli et al., 2015). In this article, we show that using object-specific models introduces the ability to classify out-of-distribution objects through a two-stage process that first aggregates the votes and then compares the prediction error on the likelihood of the observation. We devise a mechanism to aggregate information over multiple observations, and show that an embodied, enactive agent outperforms a static classifier for the object classification task. Moreover, we provide qualitative insights on how the system resolves ambiguity through the predictive model.

Additionally, we illustrate how the agent can be drawn to preferred observations through the active inference paradigm, which is crucial for object interactions such as grasping. We investigate the behavior of the latent code representing the object pose and show that the model maps similar observations to the same latent, leveraging symmetrical properties of the object structure to reduce the model complexity.

To summarize, the contributions of this article are threefold:

- We propose an object-centric model (CCN) that learns separate identity and pose factors directly from pixel-based observations through the minimization of free energy. The ensemble of CCNs for known objects form the agents generative model.
- We combine the learned identity latent representation with the likelihood of a CCN to classify objects of both seen (exact identity) and unseen (other class) categories.
- We show that through active inference, the agent can be driven toward an expected observation. We find that the agent reduces complexity in its internal model by mapping similar observations to a similar latent code.

## 2. Methods

In this section, we first discuss recent generative models for human vision, and propose our generative model for object recognition and perception. Second, we derive the free energy functional to optimize such a generative model under active inference. Finally, we present a particular instance of such a model, using an ensemble of modular deep neural networks, called Cortical Column Networks.

### 2.1. Generative Models for Vision

The Bayesian brain hypothesis finds its origin in the writings of von Helmholtz (1977), and makes the assumption that the intelligent brain reasons about the world and its uncertainty as a Bayesian process. This perspective is further formalized in terms of active inference, which posits that the brain entertains a generative model of how sensory data are generated, and functions by maximizing a lower bound on Bayesian model evidence through learning and action selection (Friston et al., 2016). Perception then boils down to inverting this model and finding the likely causes that generated the sensory data, i.e., using (approximate) Bayesian inference to compute posterior probabilities over hidden causes.

In the context of vision, this calls for inferring the causes that generate a retinal image in the case of a human, or an array of camera pixels in the case of a machine. Such a generative model should then be able to construct a scene and predict “what would I see if I looked over there” (Mirza et al., 2016). Rao and Ballard (1999) formalize a generative model for vision, through the predictive coding paradigm, by applying the underlying assumption that the external environment generates natural signals in a hierarchical manner by interacting with hidden physical causes such as object shape, texture or luminance. While their generative model considers a factorization in separate latent terms, it does not consider the influence of the observers pose and does not explicitly factorize the scene in separate objects.

**Figure 1:**
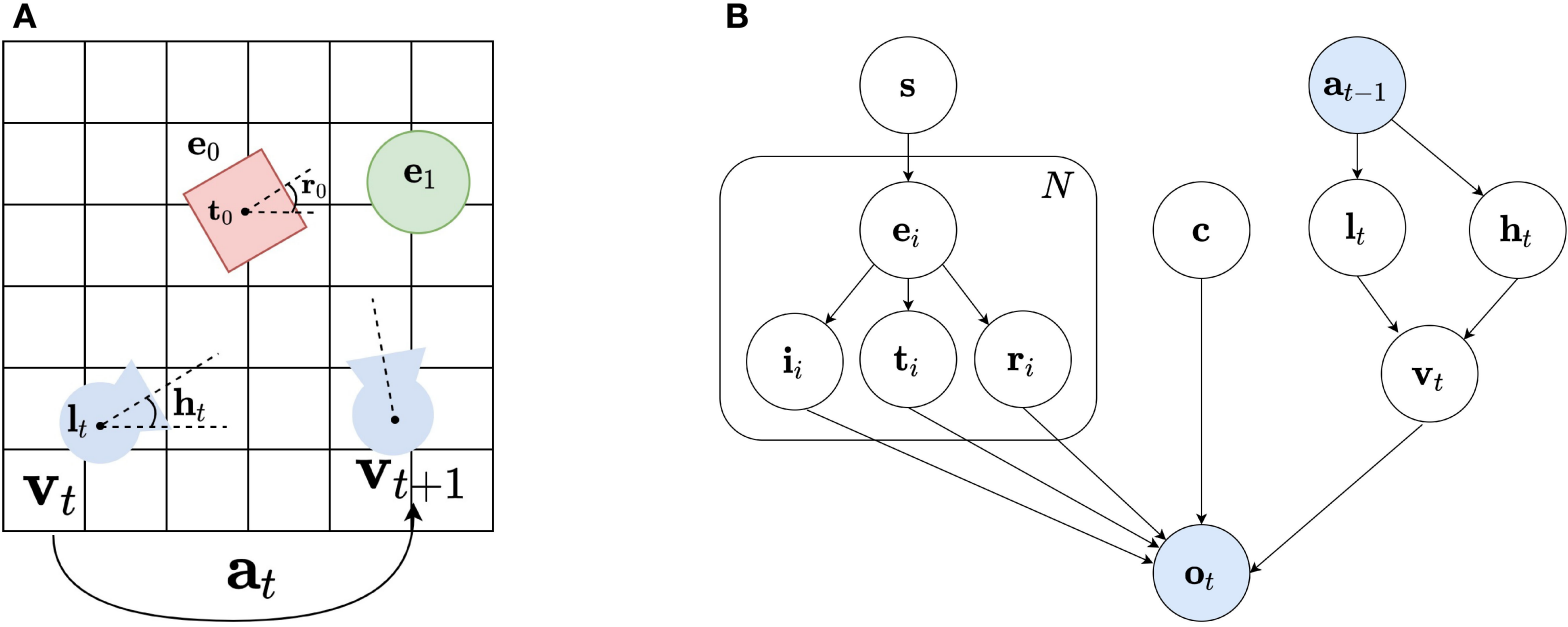
**(A)** An observer's view of the world is determined by its location **l**_*t*_ and head direction **h**_*t*_ at a timestep *t*, and the objects **e**_*i*_ in the scene, their identity **i**_*i*_, and translation **t**_*i*_ and rotation **r**_*i*_ in the world coordinate frame. The observer can take action **a**_*t*_ to move to another location. **(B)** A generative model of vision, simplified from Parr et al. (2021): starting from a scene **s**, we predict the objects or entities **e**_*i*_ one might encounter, their identity **i**_*i*_ and their placement in an allocentric reference frame defined by a translation **t**_*i*_ and rotation **r**_*i*_. Together with the context **c**, i.e., the lighting conditions, and the viewpoint **v**_*t*_ of the observer the observation **o**_*t*_ is generated. Furthermore, the observer can change its viewpoint **v**_*t*_ by taking actions **a**_*t*_ that move its location **l**_*t*_ and/or head direction **h**_*t*_. Both actions and observations are observed variables shown in blue, whereas the others are unobserved and shown in white.

A detailed generative model of human vision is proposed by Parr et al. (2021), as schematically represented in Figure 1. To predict a retinal image, one needs to know the scene and its constituent objects or entities, as well as the observer's viewpoint within that scene. This is depicted in Figure 1A: the observer's viewpoint **v**_*t*_ at timestep *t* is determined by its location **l**_*t*_ and head direction **h**_*t*_ in the scene **s**. What the observer sees are the different entities **e**_*i*_ that are described by their identity **i** and their placement in an allocentric reference frame defined by a translation **t**_*i*_ and rotation **r**_*i*_. The retinal image **o**_*t*_ is then formed from the different entities **e**_*i*_, the observer's viewpoint **v**_*t*_ together with the context **c**, e.g. the lighting conditions etc. Importantly, the observer can take action **a_t_** and move to another location in the scene, rendering vision as an inherently active, embodied process. The corresponding generative model is shown in Figure 1B, which is simplified from Parr et al. (2021), in the sense that Parr et al. (2021) also considers recursive definitions of entities, i.e., objects can again be defined as their constituent parts, and adopts a more fine grained factorization, e.g. also taking into account eye direction as separate factors.

Similar generative models can be used for learning machine vision using pixel observations (Eslami et al., 2018; Van de Maele et al., 2021b). In this case, the system is trained to make inferences about the scene **s**, given images **o**_*t*_ and corresponding absolute viewpoints **v**_*t*_. This requires massive datasets containing many views of a large variety of scenes with a number of constituent objects, typically limited to primitive shapes and colors. However, this becomes unfeasible in the real world, where the variety of objects and their arrangement in scenes yields a combinatorial explosion, and where an accurate, absolute viewpoint of the camera is often missing. Also, developmental psychology suggests that toddlers don't learn from scanning scenes, but rather focus on a single dominating object that is close to the sensors (Smith et al., 2010).

Therefore, we propose a different generative model, which is more object-centric as opposed to scene-centric. We draw inspiration from the Thousand Brains Theory of Intelligence, focused on the computational principles of the neocortex (Hawkins et al., 2019). First, we subscribe to the principle of a repetitive functional unit, i.e., a cortical column, which have basic similarity of internal design and operation (Mountcastle, 1997). Second, each such functional unit learns a model of complex objects (Hawkins et al., 2017), inferring both “what” the object is as well as “where” it is located. We model a single repetitive unit to have both the “what” and “where” information streams, this in contrast to the brain anatomy where the ventral and dorsal stream are present in separate physical areas, resulting in separate cortical columns for this function (Hawkins et al., 2019). Additionally, our model only considers a single object per functional unit rather than the numerous models a cortical column in the brain can contain.

**Figure 2:**
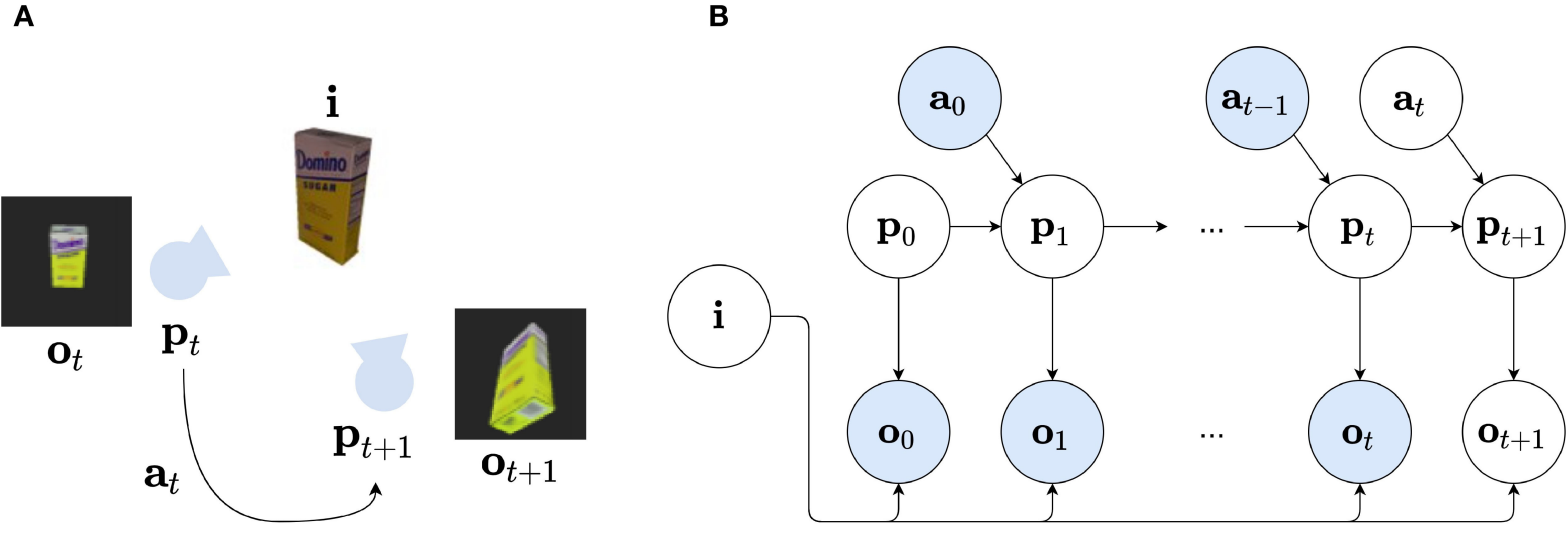
**(A)** Visual representation of the environment in which an object with identity **i** (in this case: sugar box) can be observed by a camera at a pose **p**_*t*_, relative to the object. The agent can transform this viewpoint, provided it performs action **a**_*t*+1_ to go to pose **p**_*t*+1_. At each pose, an observation **o**_*t*_ is perceived. **(B)** The Bayesian Network describing the generative model of the agent. The variable *i* represents the identity of the observed object, **p**_*t*_ represents the latent representation of the camera pose at timestep *t*. The variable **o**_*t*_ represents the sensory observation and is dependent on the identity *i* and pose variable **p**_*t*_. The current camera pose **p**_*t*_ is dependent on the previous pose **p**_*t*−1_ and action **a**_*t*−1_ of the agent. Again observed variables are shown in blue, while unobserved variables are shown in white.

Third, instead of inferring both the observer's as well as the object's poses in a global reference frame, each model learns a representation in an object-centric reference frame (Hawkins et al., 2019). Again, the agent is enactive and can move around, but instead of changing an absolute location and/or head direction, actions are now encoded as relative displacements with respect to the object at hand. This is depicted in Figure 2A: at timestep *t*, the observer captures an observation **o**_*t*_ of a certain object with identity **i**, at a certain pose **p**_*t*_ relative to the object. The observer can move around by executing action **a**_*t*_, which changes the relative viewpoint to **p**_*t*+1_.

We can formalize such an object-centric generative model as a Bayesian network, displayed in Figure 2B. We assume the agent focuses on a single object with identity **i**, and can sample different poses **p**_*t*_ by moving around by taking actions **a**_*t*_. At each timestep *t*, the object identity **i** and current pose **p**_*t*_ yield the observation **o**_*t*_. The generative model up to the current timestep *t* can then be factorized as:


(1) $$\begin{array}{l} P\left(\text{i},{\text{p}}_{0:t},{\text{o}}_{0:t},{\text{a}}_{0:t-1}\right)=\\ P\left(\text{i}\right)P\left({\text{p}}_{0}\right)\overset{t}{\underset{k=1}{\prod }}\underset{\text{Transition model}}{\underset{︸}{P\left({\text{p}}_{k}|{\text{p}}_{k-1},{\text{a}}_{k-1}\right)}}\underset{\text{Likelihood model}}{\underset{︸}{P\left({\text{o}}_{k}|{\text{p}}_{k},\text{i}\right)}}P\left({\text{a}}_{k-1}\right). \end{array}$$

The generative model hence consists of a transition model, which models how an action moves the agent to a new poses, a likelihood model that predicts the observation of an object with a given identity viewed from a given pose, and prior distributions over identity, initial pose and actions.

Crucially, we will instantiate and learn such a separate model for each and every object type. The identity variable **i** then becomes a Bernoulli variable whether or not the object at hand belongs to the object type this particular model is representing. This is interesting from a computational perspective, as it allows to train each model on a confined dataset consisting of mainly views of a single object, which improves sample efficiency, and to instantiate a new model when a new object type is “discovered”, enabling continual learning without catastrophic forgetting. To infer the object identity, we aggregate the outputs of the different models as having them casting a “vote.”

In what follows, we derive the (expected) free energy functional to infer actions for the agent to engage in active inference, and to update the model in doing so. Next, in Section 2.3, we provide more details on the actual parameterization of the model, the training mechanism and the voting scheme.

### 2.2. Active Inference

Active inference is a theoretical framework to describe the behavior of intelligent agents in dynamic environments. This theory postulates that all intelligent beings entail a generative model of the world, and act and learn in order to minimize an upper bound on the negative log evidence of their observations, i.e., free energy (Friston et al., 2016).

In order to infer beliefs about the unobserved variables, an agent needs to “invert” the generative model and calculate the posterior, which is in general intractable. Therefore, the agent resorts to variational inference, and approximates the true posterior by some tractable, approximate posterior distribution. In our case, we use an approximate posterior *Q*(**i**, **p**_0:*t*_|**o**_0:*t*_) that factorizes as follows:


(2) $$\begin{array}{l} Q(i,p_{0:t}|o_{0:t})=Q(i|o_{0:t})\prod_{k=0}^{t}Q(p_{k}|i,o_{k}). \end{array}$$

The variational free energy *F* is a quantity to describe Bayesian surprise, i.e., how much the approximate posterior and the true joint distribution differ. Given the generative model defined in Equation 1, the variational free energy *F* is then defined as:


(3) $$\begin{array}{l} \begin{array}{c} F={𝔼}_{Q\left(\text{i},{\text{p}}_{0:t}\right)}\left[\log Q\left(\text{i},{\text{p}}_{0:t}|{\text{o}}_{0:t}\right)-\log P\left(\text{i},{\text{p}}_{0:t},{\text{o}}_{0:t},{\text{a}}_{0:t-1}\right)\right]\\ =\underset{\text{complexity}}{\underset{︸}{D_{KL}\left[Q\left(\text{i}|{\text{o}}_{0:t}\right)\|P\left(\text{i}\right)\right]+\underset{t}{\sum }D_{KL}\left[Q\left({\text{p}}_{t}|\text{i},{\text{o}}_{t}\right)\|P\left({\text{p}}_{t}|{\text{p}}_{t-1},{\text{a}}_{t-1}\right)\right]}}\\ \underset{\text{accuracy}}{\underset{︸}{-\underset{t}{\sum }{𝔼}_{Q\left(\text{i},{\text{p}}_{0:t}\right)}\left[\log P\left({\text{o}}_{t}|{\text{p}}_{t},\text{i}\right)\right]}} \end{array} \end{array}$$

Hence, minimizing free energy entails maximizing model accuracy, while minimizing the model complexity, i.e., KL divergence between the approximate posterior and prior distributions. Also note that this is equivalent to maximizing the Evidence Lower Bound (ELBO) as used in variational autoencoders (Kingma and Welling, 2014; Rezende et al., 2014).

Crucially, in active inference, agents minimize the free energy not only by updating their internal model, but also by performing actions that they believe will minimize free energy in the future. However, future observations are of course not yet available. Therefore, the agent relies on its generative model to acquire expected observations over future states, and uses these to compute the expected free energy *G* for an action **a**_*t*_:


(4) $$\begin{array}{l} G(a_{t})=E_{Q(i,p_{0:t+1},o_{t+1})}[\log Q(i,p_{0\;:\;t+1}|o_{0\;:\;t},a_{t})\\ -\log P(o_{0\;:\;t+1},a_{0\;:\;t-1},p_{0\;:\;t+1},i|a_{t})]\\ \approx -\underset{\text{instrumental value}}{\underset{︸}{E_{Q(o_{t+1})}[\log P(o_{t+1})]}}\\ -\underset{\text{info gain on object identity}}{\underset{⎵}{E_{Q(i,p_{0\;:\;t+1},o_{t+1})}[\log Q(i|o_{0\;:\;t+1},a_{t})-\log Q(i|o_{0\;:\;t},a_{t})]}}\\ -\underset{\text{info gain on object pose}}{\underset{︸}{E_{Q(i,p_{0\;:\;t+1},o_{t+1})}[\log Q(p_{0\;:\;t+1}|i,o_{0\;:\;t+1},a_{t})-\log Q(p_{0\;:\;t+1}|i,o_{0\;:\;t},a_{t})]}} \end{array}$$

Here, we make two assumptions. First, we assume that the prior *P*(**o**_0:*t*+1_|**a**_*t*_)≈*P*(**o**_*t*+1_). In active inference, the agent is assumed to have prior expectations about preferred future observations (Friston et al., 2016). Because this is a prior expectation, we can leave out the conditioning on action, and it only applies on future observations. Second, we assume that the bound on the evidence is tight, and hence that the approximate posterior distributions can be used in lieu of the true posteriors, i.e., *P*(**i**|**o**_0:*t*+1_, **a**_*t*_)≈*Q*(**i**|**o**_0:*t*+1_, **a**_*t*_) and *P*(**p**_0:*t*+1_|**i**, **o**_0:*t*+1_, **a**_*t*_)≈*Q*(**p**_0:*t*+1_|**i**, **o**_0:*t*+1_, **a**_*t*_).

The result can be decomposed into three terms. The first term is the instrumental value, which values future outcomes that have a high probability under the prior distribution over preferred outcomes. Intuitively, this will yield a high value for expected observations that are similar to the preferred observation. The second term is an epistemic term that values information gain on the object identity. This means that it will result in higher values for actions that will provide more information, i.e., the expected difference between prior and posterior distributions is large. The third term is also an epistemic term that values information gain on inferring the agent's pose relative to the object. This is similar to the second term, but this time in terms of the pose latent.

### 2.3. Cortical Column Networks

In order to engage in active inference, an implementation of the generative model is needed. We choose to model the vision system as the generative model defined in Section 2.1. We use the factorization shown in Equation (1). The priors over identity, initial pose and actions are constant and are therefore not explicitly modeled. The posterior distributions of the likelihood model is defined as the distribution over the observation, when the latent variables describing identity and pose are provided. The transition model represents the relation between the pose latent at the next timestep, provided with the pose latent at the current timestep and the taken action. Finally, we amortize the inference process that infers the latent variables describing identity and pose, given an observation by an encoder model. We call the combination of a likelihood model, transition model, and encoder model for a single object category a Cortical Column Network (CCN) for this object category. In this context, amortization simply means learning a mapping from sensory input to the sufficient statistics of an approximate posterior, with a known functional form. Knowing the functional form of the posterior means the free energy objective functionals are well defined, enabling the application of standard optimization techniques (in this case Adam Kingma and Ba, 2015). This enables a generic optimization of belief distributions that underwrite active inference (Dayan et al., 1995), and can be thought of as learning to infer.

For high-dimensional data, such as pixel-based observations, designing a mapping to a latent distribution is infeasible by hand. We thus resort to deep learning to learn the likelihood and transition models directly from observation data. Additionally, we amortize the inference process and learn the encoder model jointly, similar to the approach applied in variational autoencoders (Kingma and Welling, 2014; Rezende et al., 2014).

#### 2.3.1. Model

We propose the Cortical Column Network (CCN) as basic building block of our architecture. Drawing inspiration from the Thousand Brains Theory (Hawkins et al., 2017), which promotes the modularity of cortical columns in the brain that learn predictive models of observed objects, we instantiate a separate CCN for each object type or identity. This results in a dedicated CCN for each known object type, and can be scaled to more objects by adding more CCNs. A CCN consists of three neural networks: an encoder *q*_ϕ_, a decoder *p*_ψ_, and a transition model *p*_χ_, which parameterize the approximate posterior, likelihood model and transition model introduced in Equations (1) and (2). The encoder *q*_ϕ_ has two heads that map a pixel-based observation to both a pose latent space **p**, which is modeled as a Normal distribution with a diagonal covariance matrix, and an identity latent space *i*, modeled as a Bernoulli variable. The decoder *p*_ψ_ learns the mapping from the pose latent **p** to a distribution over the observation **o**, which is modeled as a Normal distribution with fixed variance $N\left(\hat{\text{o}},I\right)$. The transition model *p*_χ_ learns to transform a sample from the pose latent **p** to a belief over the transitioned pose in latent space, also modeled as a Normal distribution with diagonal covariance matrix.

**Figure 3:**
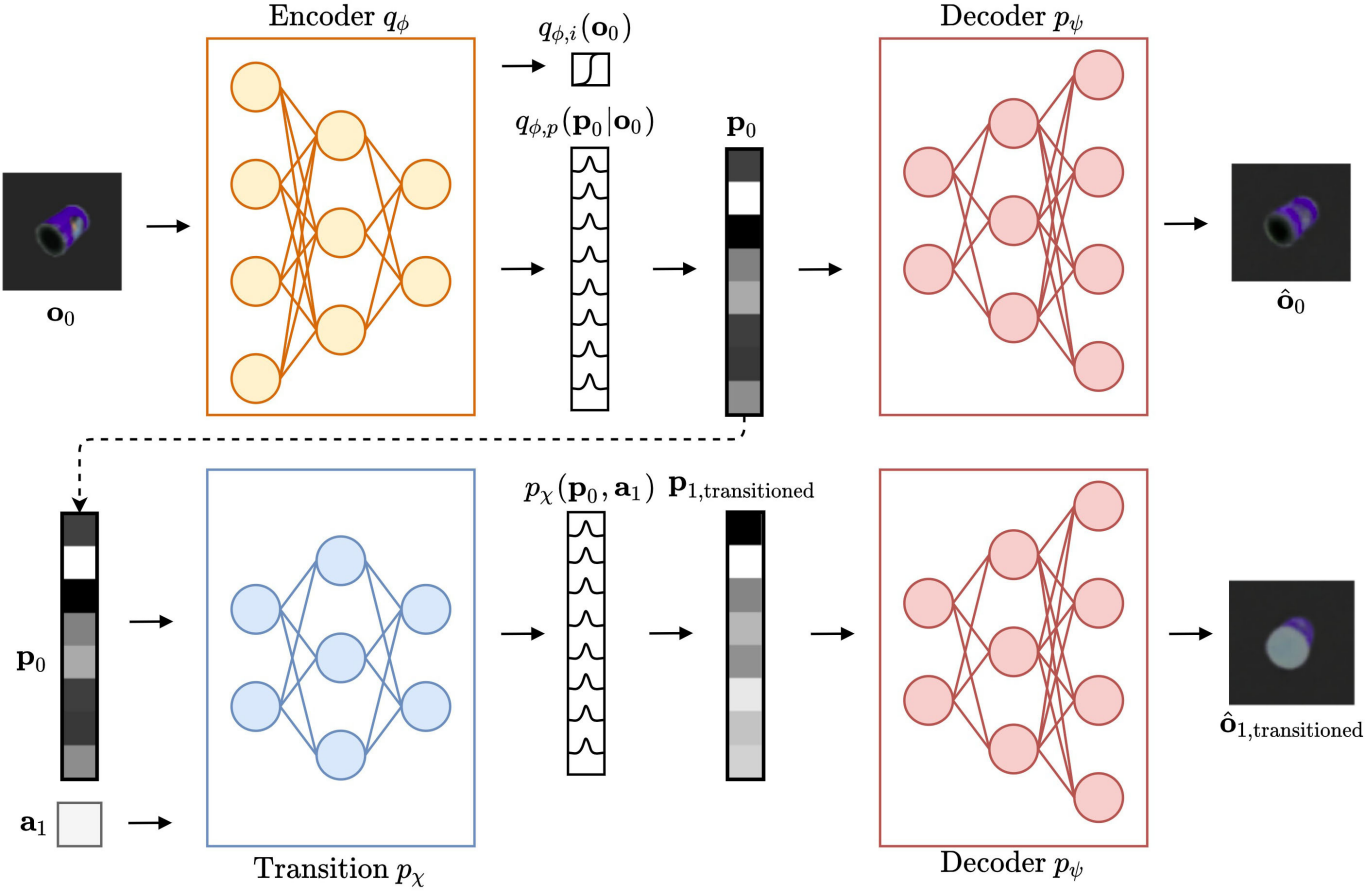
A Cortical Column Network (modeling a master chef can). In the top left, an observation **o**_0_ is provided to the encoder model *q*_ϕ_. This model predicts the distribution over identity as Bernoulli variable to be either belonging to the dedicated object category (i.e., master chef can) or not. Secondly, a distribution over the latent pose variable **p**_0_ is predicted. A sample **p**_0_ from this distribution is then decoded through the decoder *p*_ψ_ and provides the reconstruction ${\hat{\text{o}}}_{0}$. Using the transition model *p*_χ_, this pose sample is transitioned into a belief over the latent pose variable **p**_1,transitioned_, given action **a**_1_. A sample from this new belief over **p**_1,transitioned_ is also decoded into an expected view ${\hat{\text{o}}}_{1,\text{transitioned}}$.

The information flow of a single CCN is shown in Figure 3. A single CCN is dedicated to model a single object type, in this case a master chef can. An observation **o**_0_ is fed into the encoder *q*_ϕ_, as depicted in the top left corner. The belief over the identity of observation **o**_0_ is represented as a Bernoulli variable marking whether or not the observation belongs to the CCN object category. The encoder also outputs a distribution for the pose latent, from which samples can be decoded into expected observations using decoder *p*_ψ_, as shown in the top right of the figure. Finally, the bottom row illustrates the transition model *p*_χ_, which computes a belief over the pose latent **p**_1_ after taking an action **a**_1_, at current pose latent **p**_0_. Again, the decoder model *p*_ψ_ can be used to estimate observation ${\hat{\text{o}}}_{1}$ after action **a**_1_. This gives the CCN the ability to imagine “what would this object look like from here,” and to infer the best action, e.g. that minimizes the expected free energy (Equation 4). Once an action is selected, the agent moves to a new pose, obtains a novel observation **o**_1_, and the process repeats.

#### 2.3.2. Optimization

The encoder, decoder and transition neural networks for a single object are optimized in an end-to-end manner from pixel-based observations. For each object, we create a dataset $D_{i}$ from which one can sample triplets (**o**_0_, **a**_1_, **o**_1_), i.e., two images **o**_0_ and **o**_1_ together with action **a**_1_ which is the relative transform to move the camera from the initial to the next viewpoint. All viewpoints are collected such that the target object is centered in view.

The overall train procedure is given in Algorithm 1. When training the CCN for object *i*, each iteration we sample a triplet (**o**_0_, **a**_1_, **o**_1_) from $D_{i}$, as well as an observation **o**_negative_ of a random other dataset $D_{j\neq i}$. We forward all observations through the encoder model, and reconstruct ${\hat{\text{o}}}_{0}$, ${\hat{\text{o}}}_{1}$ from the pose latents **p**_0_ and **p**_1_, as well as ${\hat{\text{o}}}_{1,\text{transitioned}}$ after transitioning from *p*_χ_(**p**_0_, **a**_1_). To minimize the variational free energy as defined in Equation (3), our loss function becomes:


(5) $$\begin{array}{l} \begin{array}{c} L_{\text{FE}}=\underset{L_{\text{reconstruction}}}{\underset{︸}{\|{\hat{\text{o}}}_{0}-{\text{o}}_{0}|{|}_{2}+\|{\hat{\text{o}}}_{1}-{\text{o}}_{1}|{|}_{2}+\|{\hat{\text{o}}}_{1,\text{transitioned}}-{\text{o}}_{1}|{|}_{2}}}\\ +\underset{L_{\text{complexity}}}{\underset{︸}{D_{\text{KL}}\left[q_{\phi ,\text{p}}\left({\text{o}}_{1}\right)\|p_{\chi }\left({\text{p}}_{0},{\text{a}}_{1}\right)\right]}}\\ +\underset{L_{\text{classification}}}{\underset{︸}{\text{BCE}\left(q_{\phi ,i}\left({\text{o}}_{0}\right),1\right)+\text{BCE}\left(q_{\phi ,i}\left({\text{o}}_{1}\right),1\right)+\text{BCE}\left(q_{\phi ,i}\left({\text{o}}_{\text{negative}}\right),0\right)}} \end{array} \end{array}$$

Here, we represent the likelihood model as an isotropic Gaussian on the reconstructed pixels $\hat{\text{o}}$, which yields a mean squared reconstruction loss for the accuracy term in Equation 3, resulting in the $L_{\text{reconstruction}}$ term of the loss in Equation (5). $L_{\text{complexity}}$ in Equation (5) exactly represents the complexity term for the poses as a KL divergence term between the encoded pose distribution on the one hand, and the predicted transitioned pose distribution on the other hand. For object identity, we assume a uniform prior *P*(**i**) in Equation (3), which results in a binary cross entropy (BCE) loss term for each CCN, and we use the other object sample **o**_negative_ to contrast. These terms form the $L_{\text{classification}}$ of the loss in Equation (5). For further details on the training procedure, we refer to the implementation details in Section 3.1.

**Algorithm 1:** CCN training.

1: **for** iteration = 1, 2, … **do**
2: $\left({\text{o}}_{0},{\text{a}}_{1},{\text{o}}_{1}\right)\sim D_{i}$ ⊳ Sample observation-action pairs from the dataset of object identity i
3: ${\text{o}}_{\text{negative}}\sim D_{j\neq i}$ ⊳ Sample negative anchor
4: **p**_0_, *i*_0_ ~ *q*_ϕ_(**o**_0_) ⊳ Encode the observations and sample a pose and identity latent
5: **p**_1_, *i*_1_ ~ *q*_ϕ_(**o**_1_)
6: **p**_1,transitioned_ ~ *p*_χ_(**p**_0_, **a**_1_) ⊳ Transition the pose latent
7: ${\hat{\text{o}}}_{0}\leftarrow p_{\psi }\left({\text{p}}_{0}\right)$ ⊳ Reconstruct samples
8: ${\hat{\text{o}}}_{1}\leftarrow p_{\psi }\left({\text{p}}_{1}\right)$
9: ${\hat{\text{o}}}_{1,\text{transitioned}}\leftarrow p_{\psi }\left({\text{p}}_{1,\text{transitioned}}\right)$ ⊳ Compute the loss terms
10: $L_{\text{reconstruction}}\leftarrow \|{\hat{\text{o}}}_{0}-{\text{o}}_{0}|{|}_{2}+\|{\hat{\text{o}}}_{1}-{\text{o}}_{1}|{|}_{2}+\|{\hat{\text{o}}}_{1,\text{transitioned}}-{\text{o}}_{1}|{|}_{2}$
11: $L_{\text{classification}}\leftarrow \text{BCE}\left(q_{\phi ,i}\left({\text{o}}_{0}\right),1\right)+\text{BCE}\left(q_{\phi ,i}\left({\text{o}}_{1}\right),1\right)+\text{BCE}\left(q_{\phi ,i}\left({\text{o}}_{\text{negative}}\right),0\right)$
12: $L_{\text{complexity}}\leftarrow D_{\text{KL}}\left[q_{\phi ,p}\left({\text{o}}_{1}\right)\|p_{\chi }\left({\text{p}}_{0},{\text{a}}_{1}\right)\right]$
13: $L\leftarrow L_{\text{reconstruction}}+L_{\text{classification}}+L_{\text{complexity}}$
14: ϕ,χ,ψ←Adam(L) ⊳ Update parameters
-

Note that the distribution over the latent pose variable is modeled as a Gaussian distribution with a diagonal covariance matrix for which the parameters are learned through the optimization process. Hence, these latent dimensions do not reflect the translation and orientation parameters of an absolute pose in an Euclidean reference frame, but encode the pose in an abstract, object-local reference frame.

#### 2.3.3. Voting Over Object Identity

After training a CCN for each of the *N* known objects, our aim is to infer the object identity *Q*(**i**|**o**_0:*t*_), as a categorical distribution with *N* + 1 categories, one for each object type and an “other” category. To this end, we use a Dirichlet distribution with concentration parameters α_0:*N*_ as conjugate prior for the categorical variable. At each timestep *t*, the concentration parameters are updated as follows (Smith et al., 2022):


(6) $$\begin{array}{l} \{\begin{array}{ll} \alpha_{i,t} & =\alpha_{i,t-1}+\eta \cdot q_{\phi ,i}(o_{t})\text{, for }i<N\\ \alpha_{N,t} & =\alpha_{N,t-1}+0.5 \end{array} \end{array}$$

We initialize α_*i*, 0_ as a constant vector with values 0.1. This can be interpreted as the voting mechanism from the Thousand Brains theory (Hawkins et al., 2017), where each CCN casts a vote on whether the object in view belongs to the category it was trained on. Over time, the different votes are aggregated as collecting evidence for the different object categories. When an unambiguous view is rendered from a known object, only a single CCN, i.e., the one trained on that object category, will be active and cast a vote. However, in the case the object category cannot be distinguished from an observation, i.e., the top of a cylindrical object could be both a master chef can or a chips can, multiple votes will be cast on the different possible categories. In this case, the embodied agent can query additional views, in particular views that will provide information gain about object identity and as such minimizing the expected free energy defined in Equation 4.

In case of an unknown object, ideally none of the CCNs is active. Therefore, we add a fixed vote of 0.5 for the “other” category, which will prevail when none of the CCNs is consistently active over time. However, in practice, we find that unkown objects behave as out-of-distribution data for each individual CCN, and the predictions from the learned model are therefore unreliable. To mitigate this inherent limitation of deep neural networks, we propose an additional likelihood-based scheme for detecting the “other” category. Concretely, we look at the reconstruction error of the likelihood model to assess whether the CCN is in effect correctly modeling the object at hand. When the reconstruction error exceeds an object-specific threshold, the votes cast by the CCNs are ignored, i.e., η = 0, and only a vote of 0.5 is cast for the “other” category.

Moreover, instead of calculating the total mean squared error, we use a scaled reconstruction error. As scale factor, we choose the reciprocal of the amount of pixels in the intersection between the foreground masks of the prediction and the observation. The foreground masks are obtained by thresholding the fixed background color used in the renderings. This forces the original observation and the reconstruction to have high overlap, and increases the weight of foreground pixels for small objects.

When multiple timesteps are considered, the likelihood based threshold also considers the transition with respect to the previous observation. Concretely, when executing an action, we predict the new observation by first inferring the new pose given the previous pose and action, and reconstructing that one. Again, in order for the vote to be valid, the CCN must now have a scaled reconstruction error smaller than the thresholds for both cases.

In the case of embodied agent, the action selection process is driven through the minimization of expected free energy *G*. To infer the object identity, the prevalent term in the expected free energy *G* is the information gain term on object identity. The agent then chooses the action as follows:


(7) $$\begin{array}{l} {\text{a}}_{t+1}=\\ \underset{{\text{a}}_{t+1}}{\mathrm{argmin}}-{𝔼}_{Q\left(\text{i},{\text{p}}_{0:t+1},{\text{o}}_{t+1}\right)}\left[\log Q\left(\text{i}|{\text{o}}_{0:t+1},{\text{a}}_{t}\right)-\log Q\left(\text{i}|{\text{o}}_{0:t},{\text{a}}_{t}\right)\right] \end{array}$$

In practice, we use a Monte Carlo approximation where we evaluate this term for a number of randomly sampled actions, and select the best one. Similarly, the expectation is approximated by sampling from our models.

#### 2.3.4. Moving Toward a Preferred Observation

Once the agent has inferred the object class and its pose with respect to the object, it can also use the model to infer actions that bring the agent toward a preferred observation **o**_preferred_. This can be useful in use cases where the agent needs to inspect a particular aspect of a certain object more closely, or when the agent needs to manipulate the object and is provided with a (demonstration of a) grasp pose.

To infer the action that brings the agent toward a preferred observation, we can again evaluate the expected free energy *G*. In this case, we assume the agent already correctly inferred the object identity and pose, i.e., the information gain on these variables is low, and the expected free energy *G* boils down to maximizing the instrumental value in Equation (4), i.e., the expected error between the predicted and preferred observation. As our likelihood model in pixel-space does not necessarily reflect the perceptual difference between two images (Zhang et al., 2012), we match instead the likelihood in the pose latent space. We do this by first determining the preferred pose distribution *P*(**p**_*t*+1_) by encoding the preferred observation **o**_preferred_, and then minimizing the expected free energy with respect to the actions to match this preferred distribution, essentially computing:


(8) $$\begin{array}{l} {\text{a}}_{t+1}=\underset{{\text{a}}_{t+1}}{\mathrm{argmin}}{𝔼}_{Q\left({\text{p}}_{t+1}|{\text{p}}_{t},{\text{a}}_{t+1}\right)}\left[-\log\left(P\left({\text{p}}_{t+1}\right)\right)\right] \end{array}$$

Again using a Monte Carlo approximation, we first sample random actions, evaluate the expected free energy for all these actions with respect to the preferred pose distribution, and select the action with the lowest expected free energy. The preferred pose distribution is computed by encoding the preferred observation **o**_preferred_ using the encoder model *q*_ϕ, **p**_, whereas the expected pose distribution is acquired by transitioning the current pose latent **p**_*t*_ to an expected future pose latent using the transition model *p*_χ_.

## 3. Results

In this section, we conduct and analyze a number of experiments to evaluate our proposed approach. First we explicate the experimental setup, dataset creation, model parameterization, and training details. In a series of experiments the following research questions are addressed:

- Can a collection of CCNs be used for object classification?
- Can the ensemble of CCNs be used for detecting which object categories are out of distribution, essentially quantifying what the model does not know?
- Does embodiment improve classification accuracy as the agent can resolve ambiguity using multiple observations?
- Can a CCN for a given object category be used for object pose estimation?

### 3.1. Experimental Setup

To train our ensemble of CCNs, a dataset of different objects is required. To this end we select a subset of 33 objects from the YCB dataset (Calli et al., 2015), for which high quality triangular meshes were readily available. This set of objects is split in a known and unknown category, consisting of 26 and 7 objects respectively. For a full list of the used objects, the reader is referred to the Supplementary Materials.

For each object category of the known category, we create a dataset by rendering object meshes from this object on a uniform background. The camera poses are sampled randomly from a uniform distribution in spherical coordinates, for which the ranges are provided in Table 1. The orientation is then determined as the orientation to point the camera to the center of the object's bounding box, and randomly rotated with angle θ around the axis pointing to the object. For each object, a dataset of 10000 views is created, for which 90% is used as train data, 5% as validation data and 5% for testing.

**Table 1:** Ranges from which the absolute viewpoints are sampled in spherical coordinates in the dataset creation process.

**Variable**	**min**	**max**
azimuth	0	2π
elevation	$-\frac{\pi }{2}$	$\frac{\pi }{2}$
radius	0.10m	0.55m
θ	0	2π

We base our encoder and decoder model on the variational autoencoder architecture used in Ha and Schmidhuber (2018), where an image is first processed through a convolutional pipeline, after which a linear layer is used to transform the extracted information into the parameters of a Gaussian distribution with a diagonal covariance matrix. The decoder is the inverse of this process, where the embedding is expanded into the spatial dimensions. This result is then upscaled through a deconvolution pipeline into an expected observation. For the transition model, we simply use a multilayer perceptron network.

The encoder model *q*_ϕ_ is instantiated as a convolutional neural network that first processes a 64 by 64 RGB image with 4 convolutional layers. Each layer has a 4x4 kernel and uses a stride of 2. The layers output tensors with 8, 16, 32, and 64 channels, respectively after which they are activated through a LeakyReLU activation function with a negative slope of 0.01. The resulting representation is flattened to a 256-dimensional vector after which it is processed by two separate heads, or in other words, separate linear layers. The classification head is a linear layer, followed by a sigmoid activation function that predicts the Bernoulli variable directly. The second head predicts the mean of the belief over the pose latent by a linear layer with 8 outputs, while the variance is predicted as the softplus of the output of a third linear layer with 8 outputs.

The decoder model *p*_ψ_ is designed as the inverse of the encoder. The latent code is first expanded into a 64 dimensional vector using a linear layer, followed by a LeakyReLU (0.01 negative slope). The result is now reshaped into an image tensor that can be processed by convolutional layers. It is then processed by 2 transposed convolution layers with kernel size 6 and stride 2, after which it is fed through 2 transposed convolutions with kernel size 5 and stride 2. The output channels of these four layers are 64, 64, 32 and 16 and are followed by LeakyReLU activations with a negative slope of 0.01. Finally, a convolution layer with kernel size 1x1 and stride 1 is used to compress the channels into a 3-channel image, followed by a sigmoid to ensure the outputs are in the [0, 1]-range.

The transition model *p*_χ_ is parameterized as three linear layers that are followed by a LeakyReLU activation function with negative slope of 0.01. The first layer takes the concatenation of the pose latent code, the translation vector of the selected action and the orientation quaternion of the selected action as input, and transforms it to a 128 dimensional vector. The following two linear layers both have 256 outputs. This final output is then passed through 2 separate linear layers with 8 outputs, of which the first represents the mean of the transitioned belief and the second is passed through a softmax, which then represents the predicted variance of the belief over the transitioned pose.

The model is optimized in an end-to-end fashion using the Adam optimizer (Kingma and Ba, 2015) with learning rate 10^−4^ on the loss described in Equation (5). The separate terms in this loss function are scaled using Lagrangian multipliers (Rezende and Viola, 2018), which are inversely proportional to the gradient on the difference between the loss-term and a tolerance, to avoid posterior collapse. The multipliers for each term have an initial value and will be adapted within a specific range. The tolerances start at a fixed, low value and are updated every 500 steps. If the threshold is not reached, the tolerance is relaxed by multiplying it with a value of 1.10. This enforces the model to focus first on producing good reconstructions, and later optimize for classification and minimizing complexity. We also add a KL loss for all Gaussian outputs to standard normal to improve training stability. The values used in the optimization process are shown in Table 2.

**Table 2:** Values used in the constrained optimization mechanism (Rezende and Viola, 2018), used for training a CCN.

**Parameter**	**Initial value**	**Range**
λ_reconstruction_	80	[0, 100]
λ_reconstruction_transition_	40	[0, 100]
λ_classification_	500	[0, 1000]
reconstruction start tolerance	10	N/A
transitioned start reconstruction tolerance	10	N/A
classification start tolerance	0.01	N/A
tolerance adjust frequency	500	N/A

*Reconstruction references direct reconstruction without transitioning the latent code. The transitioned reconstruction references the reconstruction loss on the transitioned latent, and the classification terms reference the binary cross entropy terms in the loss function from Equation (5)*.

### 3.2. Classification

**Figure 4:**
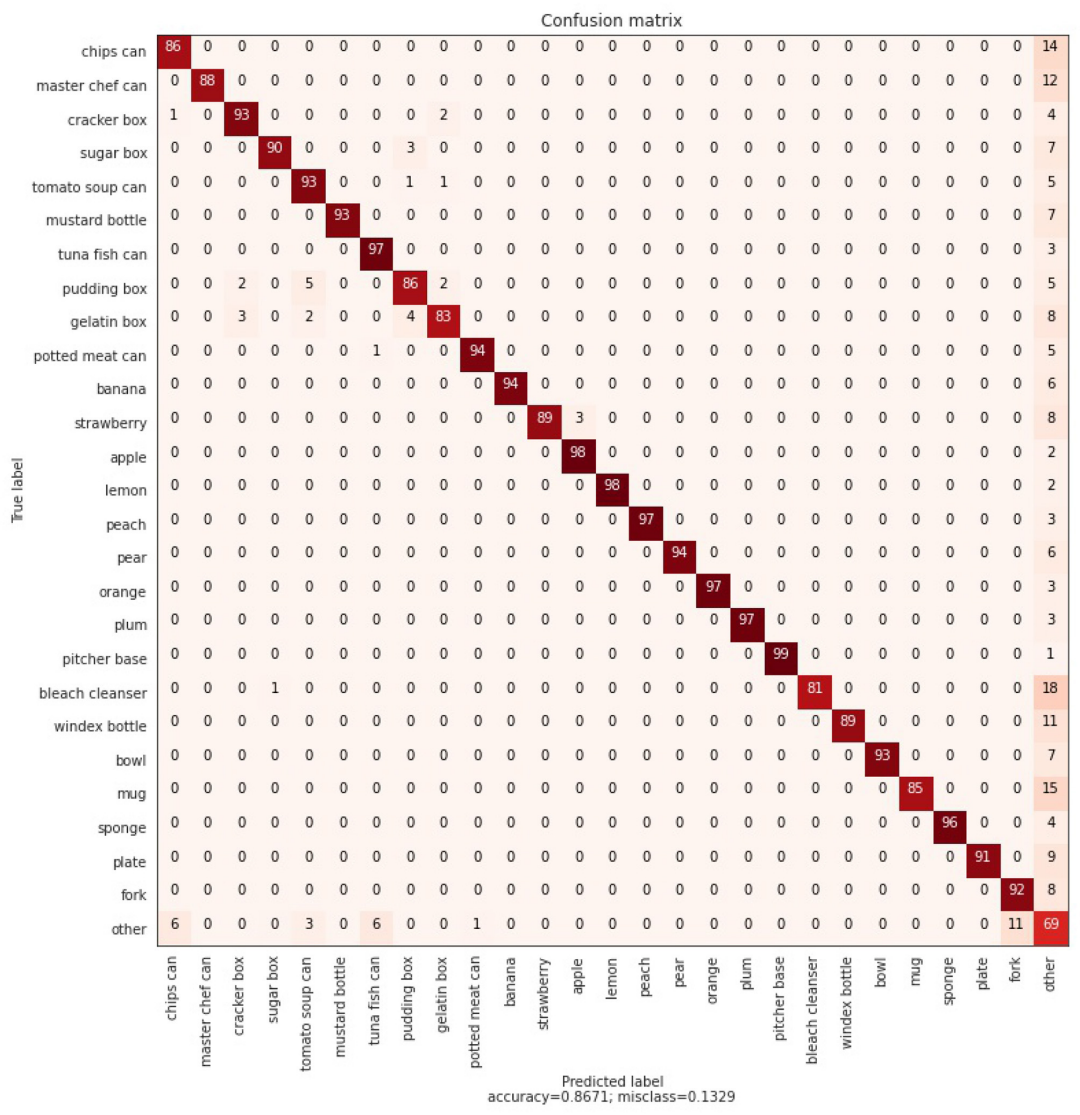
Confusion matrix, using the max of a Dirichlet distribution and a likelihood based threshold over object beliefs as described in Section 2.3. 100 examples are classified for each class of the 26 known and 7 unknown objects. Overall, an average classification accuracy of 86.71% is achieved.

First, we investigate the classification performance of our ensemble model consisting of 26 CCNs. These CCNs are each trained on a single object category, while views from the other 25 categories are used as negative anchors. The 26 object categories are listed in the confusion matrix, shown in Figure 4. First, we evaluate the performance of classifying a single observation, followed by an experiment in which an embodied agent can query multiple observations sequentially.

#### 3.2.1. Static Agent Classification

To investigate the classification performance of a static agent, we provide the agent with a single observation. We address whether an ensemble of CCNs can be used for accurate object classification. Additionally, we investigate to what extent our approach can accurately detect when an object is out of distribution, i.e., the object does not belong to a category previously seen by the agent during training.

For each object category, 100 samples are randomly sampled from the test for classification, and all unknown objects are clustered in an “other” category. As described in Section 2.3, each CCN votes for the known category it was trained on, provided that the reconstruction likelihood is within a predefined threshold. We empirically determine the threshold for each category by looking at the reconstruction errors of train-set observations, and scale the 95% quantile value by a factor 1.1, to remove outliers. This results in a high classification performance while still being able to detect more novel objects.

**Figure 5:**
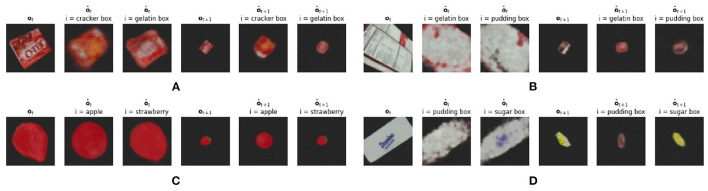
Ambiguities found in the ensemble CCN classifier. From left to right: initial observation **o**_*t*_ provided to the agent. The reconstruction ${\hat{\text{o}}}_{t}$ predicted using the expected model. The reconstruction ${\hat{\text{o}}}_{t}$ predicted using the correct model. The chosen next observation **o**_*t*+1_ through the minimization of expected free energy *G*. The expected observation ${\hat{\text{o}}}_{t+1}$ using the selected model. The expected observation ${\hat{\text{o}}}_{t+1}$ using the correct CCN model. **(A)** The gelatin box. **(B)** The pudding box. **(C)** The strawberry. **(D)** The sugar box.

We show the confusion matrix for the static agent in Figure 4. An average classification accuracy of 86.71% is achieved. The confusion matrix shows that the main source of errors is due to the CCNs not being confident enough on the reconstruction and the “other” vote wins. We also see that in some cases there is some confusion between similar shaped objects, i.e., between “pudding box,” “cracker box,” and “gelatin box.” We hypothesize (see Section 3.2.2) that querying more observations of the same object will adjust the vote for the correct object category, and after multiple observations the agent will resolve these issues. We qualitatively investigate these difficult samples, as is shown in Figure 5. This figure shows ambiguous observations that are incorrectly classified by the ensemble of CCNs. It can be observed that the reconstruction from both the (wrongly) chosen model and the correct model are very similar. For example for the strawberry and the apple, a large red circle is reconstructed. It is thus difficult to accurately predict the object class.

Figure 5 also shows the expected next viewpoint that would be encountered if an action minimizing expected free energy *G* was performed. The latent code for both potential object categories is acquired through both the correct and incorrectly chosen transition models and an imagined view can be acquired using the respective decoders. Clearly, these selected observations are more easily distinguishable and thus enforce our hypothesis that embodiment will aid in the correct classification of three dimensional objects.

Alternatively, we could also train a single classifier using the same amount of parameters as the ensemble of CCNs which we expect to achieve similar classification accuracy to the ensemble of CCNs. However, due to the inherent nature of the model design, it would be unable to estimate when objects from an unseen category occur and this model would not have the flexibility to add new modules when novel objects are encountered.

#### 3.2.2. Embodiment and Aggregating Votes

The previous section showed some clear disadvantages using a static agent: ambiguities can not be resolved, nor can information from previous observations be used to make a more confident, and more reliable decision. In the following experiment, we investigate whether classification accuracy improves as the agent is embodied and can actively query novel viewpoints. At each timestep, the agent randomly samples 100 action candidates and evaluates the expected free energy, i.e., to maximize the information gain on object identity as stated in Equation (4). This action is then executed and a novel observation is provided to the agent, which updates the Dirichlet concentration parameters and the process repeats.

**Figure 6:**
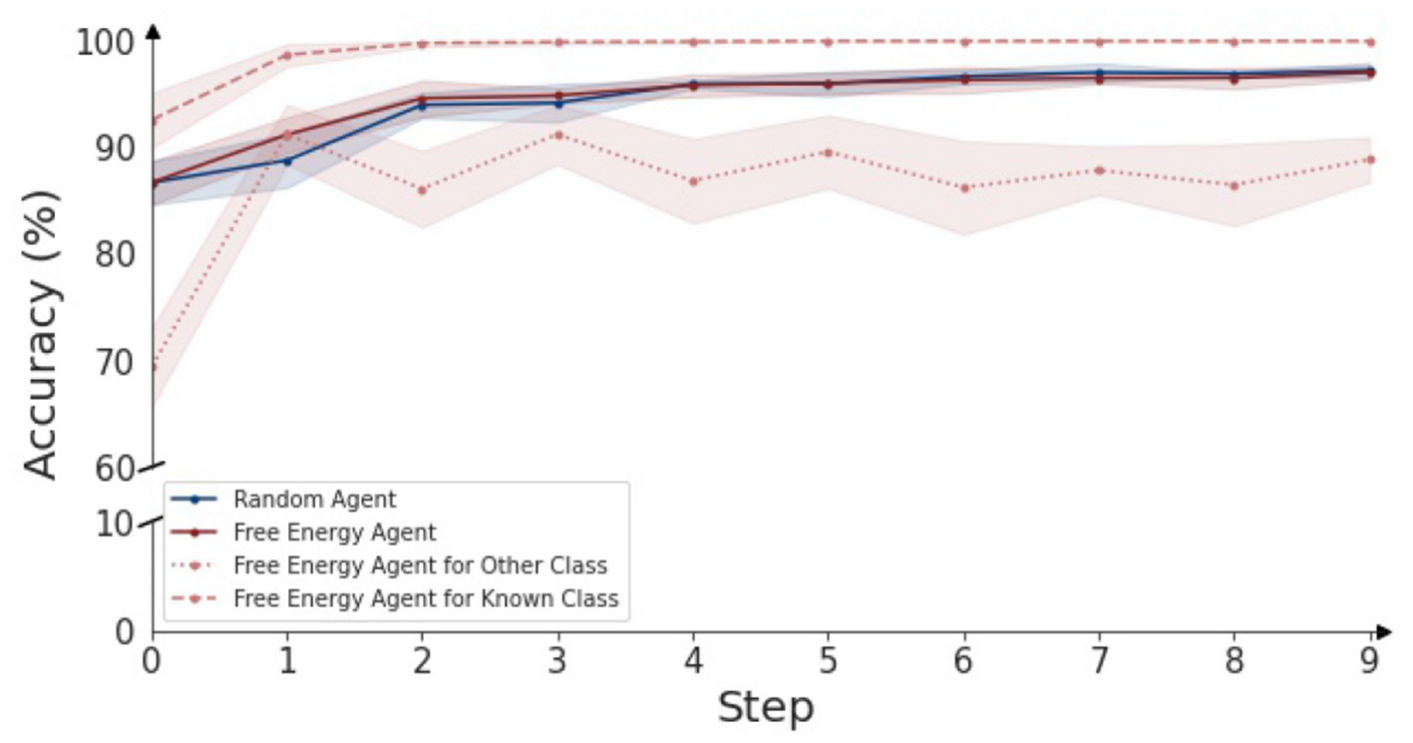
Classification accuracy over time for an embodied agent, driven through the active inference paradigm. The agent is provided with different objects in random poses to classify, accuracy over a duration 10 steps is plotted. For each object category, 5 splits of 20 observations are classified and are used to visualize the 95% confidence bounds. The graph indicates classification accuracy over time for objects of the 26 known and 7 unknown objects. The red line represents the accuracy for the free energy agent, while the blue line represents the accuracy for the random agent. For the active inference agent, the distinction is made between the known and unknown objects: the dotted red line indicates the classification accuracy for objects of the 7 unknown object categories and the dashed line indicates the classification accuracy for the 26 known objects.

In Figure 6, the classification accuracy of an active inference driven agent over time is shown for different datasets. When testing the agent only on the known classes (dashed line), the agent can immediately resolve ambiguities and the performance reaches 100% after two steps. When only considering objects from the 7 “other” categories (dotted line), the classification accuracy starts at a lower value of around 70% (as can also be seen in the confusion matrix in Figure 4), and over time reaches an accuracy of 85%. Finally, the red line shows the classification accuracy for all objects combined (26 known and 7 unknown objects). The performance rises from 87 to 97% after nine steps. The full confusion matrix for each different step can be found in the Appendix.

It can be observed that the accuracy for the known classes only increases. This is attributed to the Dirichlet information aggregation scheme. As more information is acquired, the votes and evidence for certain object categories becomes more overwhelming. In contrast, accuracy for the other category clearly gains information after a single timestep, but then fluctuates between 80 and 90%. As described in Section 2.3, the other category is mainly detected by the second reconstruction-based phase of the classification pipeline. This phase considers the current observation, and the transition given the previous observation, the window of information is thus two timesteps, and therefore no classification performance increase is found after more than two steps.

As a comparison baseline, we evaluated the embodiment using a random agent, i.e., the next viewpoint is randomly selected instead of using free energy minimization. The accuracy this random agent realizes, is indicated by the blue line in Figure 6. The performance of the random agent is on par with the active inference agent. We also observe that the ratio of informative views with respect to ambiguous views is high. Recall from the confusion matrix, the correct object identity can be inferred in over 80% in the first step of the (randomly) sampled views. Hence, it is to be expected that providing a random additional view provides the necessary information to get the correct classification, and the free energy agent has only a small margin to improve upon. We expect the gap between the free energy agent and a random agent to become larger in the case where more ambiguous viewpoints are present, as the free energy agent will avoid those as evidenced by Figure 5.

### 3.3. Pose Estimation

Next, we evaluate to what extent CCNs can be used for object pose estimation, given a desired view. First, we qualitatively evaluate the object pose estimation for different objects. At each timestep, the agent samples 1,000 random actions and calculates the instrumental term of expected free energy *G* as described in Section 2.3.4. Again, the agent selects the action that minimizes the expected free energy and queries a new observation.

**Figure 7:**
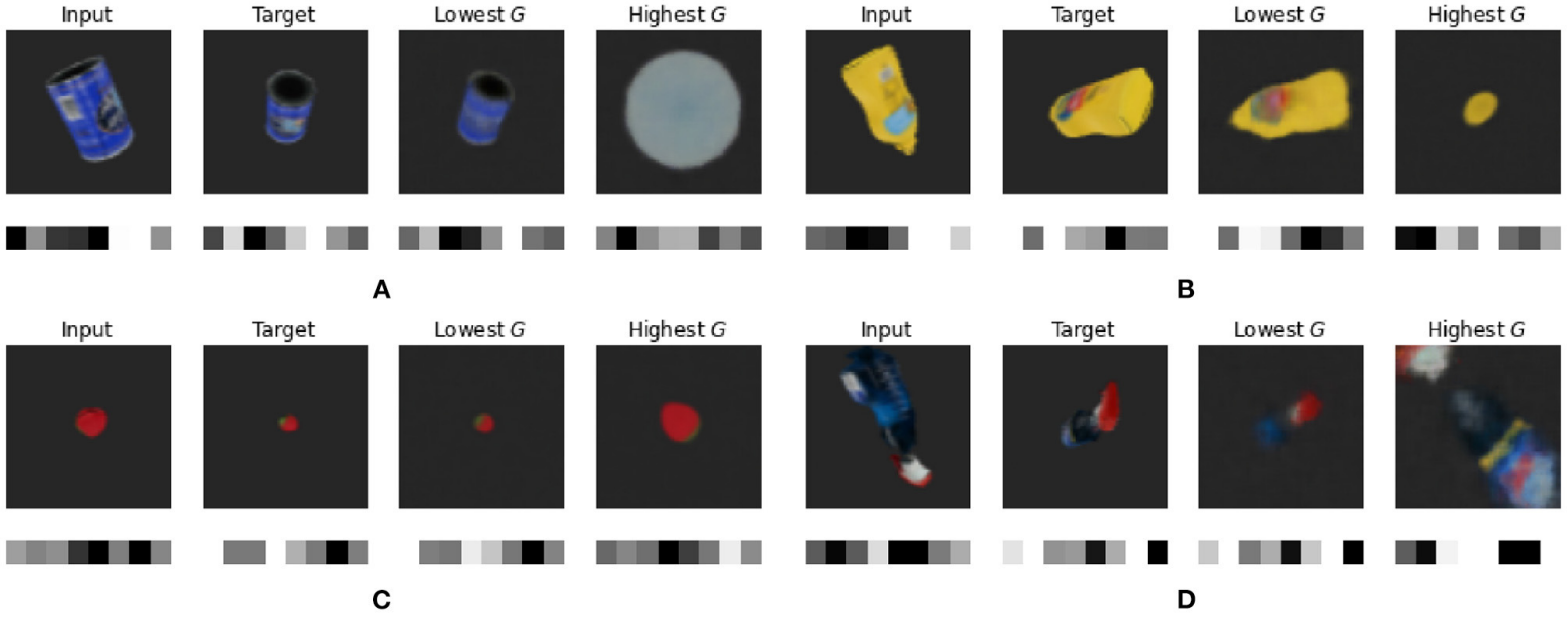
Qualitative results for object pose estimation for **(A)** master chef can, **(B)** mustard bottle, **(C)** strawberry, and **(D)** windex bottle. The first column shows the input of the model with the mean latent code shown below. The second column shows the target observation along with its mean latent code. The third column shows the imagined observation and the transitioned latent code for the action with the lowest expected free energy *G*, while the final column shows the imagined observation and latent code for the action with the highest expected free energy *G*.

In Figure 7, we plot the input and target views, as well as the predicted viewpoints with the best (lowest) and worst (highest) expected free energy *G* for master chef can, mustard bottle, strawberry and windex bottle. Below each observation the inferred or predicted latent code is shown. It is clear that the latent code is similar for matching the observations, while having the additional benefit that it does not suffer from the typical issues with MSE such as the scaling issue for pixel-wise errors.

However, when we quantitatively evaluated the resulting poses, we noticed that the absolute pose error in Euclidean space was often way off, despite similar reconstructions. To further inspect this, we plot the expected free energy landscape for varying azimuth and elevation for the predicted target pose, as well as the initial, target and selected pose.

**Figure 8:**
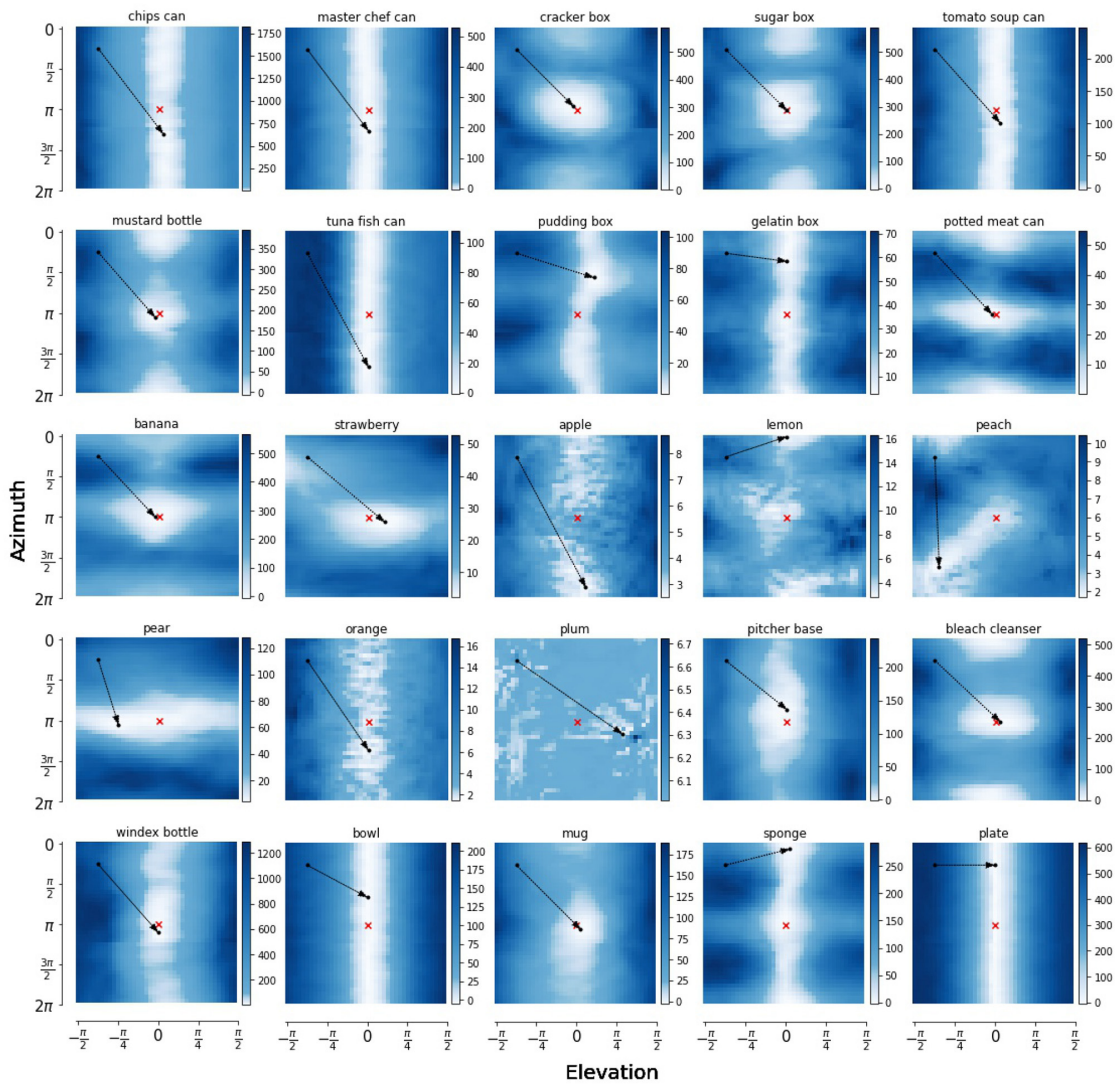
Heatmaps of the expected free energy *G* for reaching a target observation, marked by the red x. The pose parameters radius and angle θ around the z-axis of the camera are kept in a fixed position, while varying the azimuth and elevation angle over their full range in 40 linearly spaced points in each dimension. The initial point and the point with lowest expected free energy *G* are marked by black dots. The arrow head points at the point with lowest free energy, starting at the pose of initial observation.

In Figure 8, we show heatmaps of expected free energy *G* for 25 objects from the YCB dataset in the pose estimation scheme. The exact pose can be represented by four degrees of freedom: azimuth, elevation, radius and axis angle θ. We vary two of these dimensions while keeping angle θ and radius fixed and plot the expected free energy landscape for the agent to reach a target observation, marked by the red cross. As indicated by the figure legend, the lightly colored areas are more desired by an active inference agent as they have a lower expected free energy. The red cross marks the preferred state of the agent, and the black dots show the initial observation and the point with lowest expected free energy.

For some objects, such as banana and mug, there is a clear global minimum in the expected free energy landscape, and the pose estimation is quite accurate. However, for other objects, such as sugar box, mustard bottle and bleach cleanser, there are multiple local optima, or the landscape might even be invariant to the azimuth axis, as is the case for a lot of the can and box objects, the bowl and plate. These areas with low free energy are aliased areas, where the symmetry of the object surfaces. This shows how our model has actually learned various object symmetries, and learns to map different aliases with similar pixel observations onto the same point in pose latent space.

**Figure 9:**
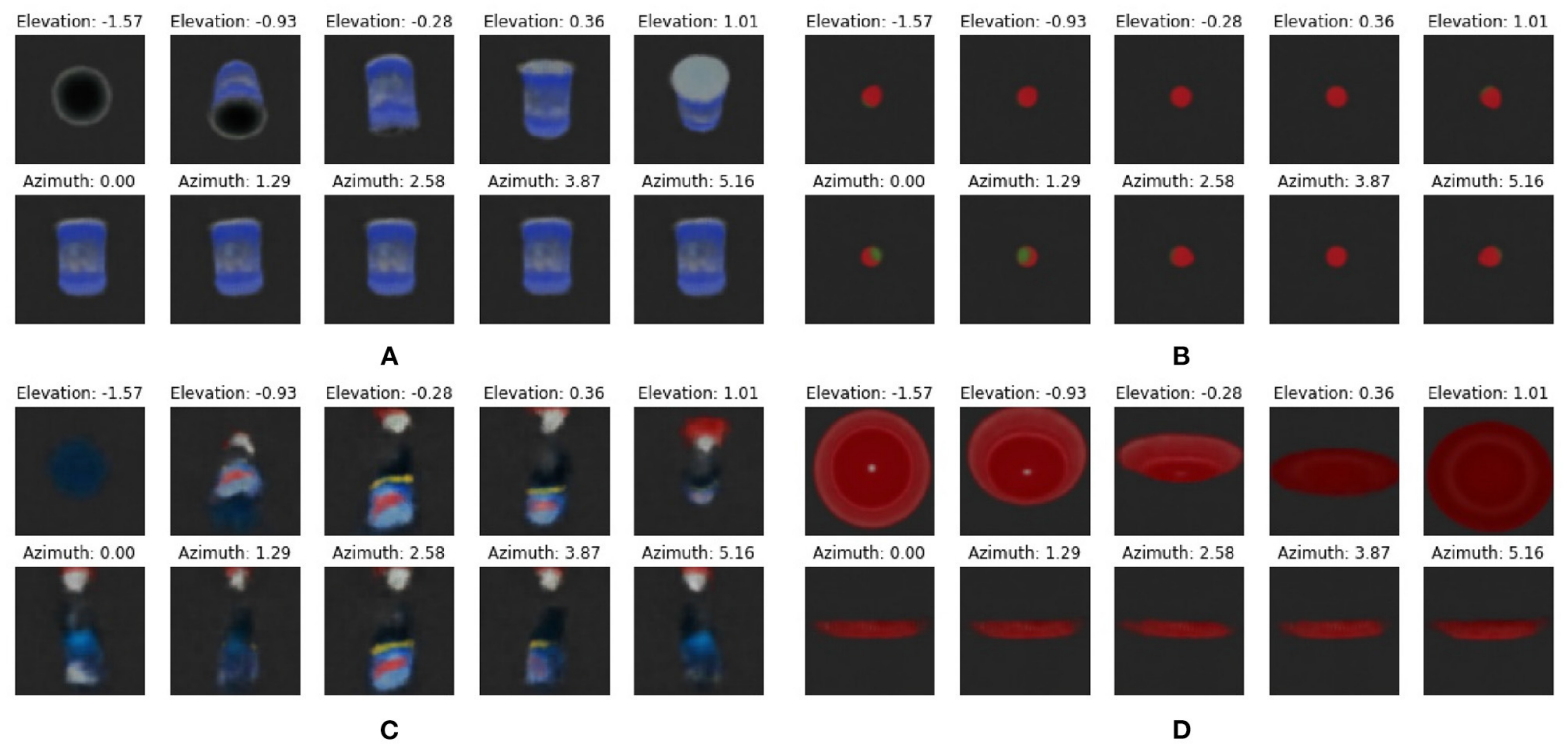
Imaginations generated from CCN models of **(A)** master chef can, **(B)** strawberry, **(C)** windex bottle, and **(D)** plate. We vary a single dimension while keeping the other three at a fixed position. The top row shows a varying elevation and the bottom row shows a varying azimuth, within the full range as defined in Section 2.3.

This can be viewed more clearly for imaginations generated while varying one dimension of these plots. Figure 9 shows imaginations of a varying azimuth or elevations while keeping the three other dimensions fixed. In the heatmap of the master chef can, it can be observed that varying the azimuth results in the same expected free energy, while this differs for changing the elevation. Figure 9A shows this more clearly as all reconstructions of a straight can are identical. The model did not learn to reconstruct the exact contents of the can label, but blurs this as an average of the whole can. Similarly for Figure 9D, a different azimuth will show a horizontal plate, and no difference can be found. In contrast, the strawberry has a fairly localized expected free energy minima, which can be attributed to the position of the green on the strawberry head. For this reason, the different orientations can be differentiated. The same can be found for the windex bottle, where the objects inherent asymmetry results in a clearer loss landscape.

## 4. Discussion

In this article, we proposed a method for learning object-centric representations in an unsupervised manner from pixel data. We draw inspiration from recent theories in neuroscience, in particular an active inference account of human vision (Parr et al., 2021) and the Thousand Brain Theory on intelligence (Hawkins et al., 2017). This leads to a modular architecture, where each model separately learns about an object category and a pose in an object-centric reference frame. We called our modular building block a Cortical Column Network, referring to the cortical column structures in the neocortex which (Hawkins et al., 2017) hypothesize also model objects in a local reference frame. However, despite the similarities, it is important to note that there are also important differences with how biological cortical columns are supposed to work in the Thousand Brains theory. For instance, each cortical column in the neocortex processes a distinct, small sensory patch, whereas our CCNs all work on the same, full resolution camera input. Moreover, each cortical column is hypothesized to model and vote for a larger number of object categories, which yields a more scalable processing architecture and sparse object representations. Finally, we also note that as the “what” and “where” information stream are located at distinct areas in the brain, this information is also processed through separate cortical columns. These and other aspects are not treated in our current CCN architecture, and it remains an exciting research direction to investigate to what extent artificial agents should mimick biologically plausible architectures and processing methods. For example, the representation of multiple internal models, or hypothesis for the sources of sensory information, has been explored in the context of birdsong and social exchange in the auditory domain (Isomura et al., 2019). Again, the basic idea is that multiple hypotheses are entertained and the model with the highest evidence contributes more to the posterior beliefs (i.e., Bayesian model average) about latent states or codes. Embedding our CCNs within the active inference framework enabled us to integrate both model learning and action selection under a single optimization objective. It would be interesting to investigate to what extent biological cortical columns could also be modeled to engage in active inference to produce motor commands.

Having a repertoire of object-specific cortical columns, who can “vote” or “compete” to explain sensory input, can be understood from a number of perspectives. The thousand brains perspective is closely related to mixtures of experts, of the kind found in MOSAIC architectures for motor control (Haruno et al., 2003). Perhaps most generally, it can be regarded as a simple form of Bayesian model averaging (Hoeting et al., 1999). In other words, each cortical column builds a posterior belief about the attributes of an object, under the hypothesis or model that the object belongs to a particular class. The evidence for this model is then used to form a Bayesian model average over object attributes. A Bayesian model average simply marginalizes over the models by taking a mixture of posteriors under each model, weighted by the evidence for the respective models (usually, a softmax function of variational free energy).

Visual information is processed according to two major fiber bundles in the brain. It is hypothesized that these two fibers process separate aspects of the observed visual stimulus. The observed object identity is processed through a ventral pathway, and the objects location is processed through a specialized dorsal pathway (Grobstein, 1983). We found that using a ensemble of CCNs, a high accuracy classifier can be built combining both a ventral (“what”) stream to infer object identity, and a dorsal (“where”) stream inferring an object pose, and predicting other viewpoints. Crucially, we showed that an enactive, embodiment agent is required both to train such a system unsupervised, by collecting a dataset of viewpoints of each object, as well as to make correct inferences, and resolve ambiguity in the observation. In this work, we decoupled the data collection and the inference phase, and trained on a dataset of a relatively large number of randomly queried poses. Under active inference, one can also model the inference process over model parameters (Friston et al., 2016), and actively sample views that one expect to maximize information gain for the training process. It is worth investigating whether the model can be trained more efficiently, by driving the agent to the most informative view using the information gain on model parameters in the expected free energy functional. Information gain on model parameters is, in the active inference literature, called “novelty”, while information gain on latent states or attributes is associated with “salience” (Schwartenbeck et al., 2019).

We also investigated the pose estimation properties using the dorsal (“where”)-stream of our model. We showed that we are able drive the agent's actions toward a preferred, target pose by providing the corresponding observation. While we showed that the agent is indeed able to find a viewpoint with a similar observation, we also found that a lot of alias viewpoints exist in the latent space, due to object symmetries on the one hand, and the lack of sufficient visual details captured by the model, i.e., to disambiguate the front or back label of an object. However, we argue that in the case of robotic manipulation, this level of performance would already be sufficient for basic manipulation tasks such as grasping or pushing. Nevertheless it remains an important area of future work to find models that are able to capture and encode the required level of (visual) detail.

In addition there are still a couple of limitations in our current setup that might be addressed in future work. For example, our models currently learn the representation for a single object instance of an object category. In simulation, there is no variation between multiple instances of the mustard bottle, however, in real life the label can be attached crooked, or some markings can be present on the object. The current CCNs do not generalize to perturbations of the objects let alone other objects belonging to the same category, i.e., a coffee mug with a different height or color. It is worth investigating whether a single CCN can contain representations of different instances of a more general object category. Also note that our current CCN models are trained from scratch for each novel object category. Hence, a lot of overlapping information has to be relearned. Learning to re-use information would yield CCNs that are closer to the thousand brains theory as the cortical columns in the brain also reuse information (Hawkins et al., 2019). In order to re-use information learned by the CCNs, a potential extension would be to share weights between all CCNs for part of the layers, or devise a more hierarchical approach modeling part-whole relationships (Hinton, 2021).

Finally, our CCN models only encode egocentric representations in an object-local reference frame. In order to model a whole scene or workspace, the agent will need to map these egocentric poses into an allocentric reference frame (Parr et al., 2021). This would enable the agent to build a cognitive map of the workspace, inferring for each object an allocentric pose in the workspace, and “navigate” from one object to another. This would then give rise to a hierarchical generative model, mapping the world and its constituent objects using the same priniciples as simultaneuous localization and mapping (Safron et al., 2021).

###  Related Work

In previous work, we built an artificial agent that learned such a generative model from pixel data, inferring beliefs about a latent variable representing the scene **s**, given image observations **o_t_** from absolute viewpoints **v**_*t*_ (Van de Maele et al., 2021b). Similar to a Generative Query Network (GQN) architecture (Eslami et al., 2018), this approach requires a huge train set of different scenes, with a limited set of constituent objects, in order to learn valid scene representations. The representations from this model encode all present objects and their relative pose with respect to the global allocentric reference frame. As a result, this lacks a factorization of different objects, and does not scale to a large number of objects present in the scene.

Most object-centric representations stem from the seminal work Attend Infer Repeat (AIR) by Eslami et al. (2016), where an image of a scene is factorized as a collection of latent variables separately describing the what and where parameters of each object. These variables are recurrently predicted, and can thus be scaled to an arbitrary amount of objects in the scene. AIR considers a static observer looking at a single observation. Burgess et al. (2019), proposed MONet, which learns the decomposition in an unsupervised end-to-end fashion. They also learn a structured representation describing each object. IODINE (Greff et al., 2019) also learns a joint decomposition and representation model but requires a fixed amount of slots that can be filled in by separate objects. Other work focuses on dynamic scenes by adding a temporal component (Kosiorek et al., 2018). They do this by adding a propagation module for objects from previous timesteps, and a discovery module that detects novel aspects. Other follow-up works tackle the scalability problem (Crawford and Pineau, 2020; Jiang et al., 2020) by predicting segmentation masks directly. Lin et al. (2020) combine the scalability and temporal works, and add multimodality in the model through sampling in multiple steps. More recent work also considers three dimensional scenarios (Chen et al., 2021) with primitive shapes such as cubes or spheres where a generative query network (Eslami et al., 2018) is used as a rendering module for objects separately.

Similar to these models, we also make the separation in a what and where latent code. However, instead of forcing the factorization in a single latent space, we factorize on a model level, which results in a modular CCN model, where each CCN can focus on a single object type. While all mentioned models acquire impressive results on either static or dynamic observation data, none of these models consider an embodied, enactive agent to improve perception, which we believe to be crucial for intelligence.

An upcoming type of models are the implicit representation models that learn three dimensional structure explicitly in the model weights (Mescheder et al., 2019; Park et al., 2019; Mildenhall et al., 2020; Sitzmann et al., 2020). Neural Radiance Fields (NeRF) (Mildenhall et al., 2020), can learn complex object geometry by directly optimizing color and opacity values when conditioned by the coordinate and orientation of a point in the three dimensional space. This is optimized end-to-end directly from observation by casting rays from the camera pose and inferring sampled points on this ray. In follow-up work, different ways to optimize these models real-time by selecting key observations and strategic sampling of rays were found (Sucar et al., 2021). Similar to the implicit representation models, we learn to reconstruct object observations from a different set of observations. While the reconstruction detail of these models is impressive, these models lack an inverse model to infer poses or object categories.

A popular brain-inspired paradigm for unsupervised representation learning is predictive coding (Rao and Ballard, 1999). This mechanism works by hierarchically estimating the input and only propagating the error. This way, the lower levels of the hierarchy focus on smaller details of the observation. This work has also been used to separate the “what” and “where”-information streams (Rao and Ruderman, 1999). The predictive coding paradigm can be recast as active inference when using distributions over the predictions, rather than point estimates and when actions can be inferred to lead the artificial agent to a preferred goal state (Jiang and Rao, 2021).

The proposed approach in this article is also closely related to the object pose estimation research domain. These methods typically try to estimate the object pose directly as a 6 dimensional vector representing both the translation and orientation with respect to an absolute reference frame. Within the taxonomy provided in the survey paper by Du et al. (2021), our method could best be classified under the template-based label: given an observation, the model tries to find the pose that best matches one of the pre-defined labels. In this case, a trained CCN amortizes the process of finding the exact template through encoding the observation. The most closely related approaches use convolutional neural networks to directly estimate the object pose, and are pretrained on a set of labeled data which can be considered the templates (Do et al., 2018; Xiang et al., 2018; Liu et al., 2019). While these approaches acquire high accuracy results, they are trained supervised with a labeled dataset. In contrast, our approach is trained unsupervised from sequences of observations an enactive agent could perform, enabling our model to learn in arbitrary environments. This also has the corollary that the learned pose is in a non-interpretable latent space and can not be decomposed in an explicit translation and orientation.

The active inference (Friston et al., 2016) framework has also been previously adopted for describing generative models for active vision (Parr et al., 2021). In prior work, this framework has been shown to drive intelligent agents for visual foraging (Mirza et al., 2016; Heins et al., 2020), or for creating fovea-based attention maps to improve perception accuracy (Dauc, 2018). However, these works typically work with simpler, human engineered generative state space model, whereas in our case, the models are learned end-to-end from pixels. Different works also combine active inference with deep learning for learning state spaces directly using pixel-based observations (Çatal et al., 2020b; Fountas et al., 2020; Mazzaglia et al., 2021), but focused more on pixel-based benchmarks for reinforcement learning.

## 5. Conclusion

In this article, we proposed a novel method for learning object-level representations, drawing inspiration from the functional properties of the dorsal and ventral stream in the human neocortex. We made a separation on an object level, and create a basic building block for learning representations, which we coin a Cortical Column Network or CCN. We first described a generative model that casts vision as making inferences about an object pose and identity. For this generative model, we derived the (expected) free energy functional, which is used for both optimizing the model parameters as well as driving the agent actions toward desired poses or gaining information for better inference.

We showed that an ensemble of CCNs can be used for accurate object classification. By aggregating CCN predictions as “votes” in a Dirichlet distribution, we are able to correctly identify all known objects, while at the same time also being able to detect never seen before objects as an “other” category. We showed how an enactive, embodied agent improves the classification accuracy over time, by actively sampling novel observations that reduce ambiguity. We also investigated the ability of a CCN for reaching a preferred pose, given a target observation. We qualitatively evaluated how indeed the agent moves toward a matching observation. In addition, we explored the expected free energy landscape, showing that our models learn an abstract latent space for encoding pose in an object-local reference frame, exploiting object symmetries.

We believe that developing algorithms for learning in enactive, embodied agents is key to build artificial intelligent agents. To do so, we should rather inspire ourselves by the domains that study such embodied agents, i.e., behavioral psychology, biology and neuroscience, rather than only limit ourselves to the domain of artificial intelligence. We hope this work takes a small step in that direction.

## Data Availability Statement

The raw data supporting the conclusions of this article will be made available by the authors, without undue reservation.

## Author Contributions

TVa and TVe conceived and performed the experiments and worked out the mathematical basis for the experiments. TVa, TVe, OÇ, and BD contributed to the manuscript. BD supervised the experiments. All authors contributed to the article and approved the submitted version.

## Funding

This research received funding from the Flemish Government (AI Research Program). OÇ was funded by a Ph.D. grant of the Flanders Research Foundation (FWO). Part of this work has been supported by Flanders Innovation & Entrepreneurship, by way of Grant Agreement HBC.2020.2347.

## Conflict of Interest

The authors declare that the research was conducted in the absence of any commercial or financial relationships that could be construed as a potential conflict of interest.

## Publisher's Note

All claims expressed in this article are solely those of the authors and do not necessarily represent those of their affiliated organizations, or those of the publisher, the editors and the reviewers. Any product that may be evaluated in this article, or claim that may be made by its manufacturer, is not guaranteed or endorsed by the publisher.
